# Comprehensive Analysis of Porcine *Prox1* Gene and Its Relationship with Meat Quality Traits

**DOI:** 10.3390/ani9100744

**Published:** 2019-09-29

**Authors:** Chao Dong, Xiying Zhang, Kaiqing Liu, Bojiang Li, Zhe Chao, Aiwen Jiang, Rongyang Li, Pinghua Li, Honglin Liu, Wangjun Wu

**Affiliations:** 1 Department of Animal Genetics, Breeding and Reproduction, College of Animal Science and Technology, Nanjing Agricultural University, Nanjing 210095, China; 2016105025@njau.edu.cn (C.D.); 2018105030@njau.edu.cn (X.Z.); 2015205001@njau.edu.cn (K.L.); libojiang12@126.com (B.L.); 2015105077@njau.edu.cn (A.J.); 2015105013@njau.edu.cn (R.L.); Lipinghua718@njau.edu.cn (P.L.); liuhonglin@163.com (H.L.); 2 College of Animal Science and Veterinary Medicine, Shenyang Agricultural University, Shenyang 110866, China; 3 Institute of Animal Sciences & Veterinary, Hainan Academy of Agricultural Sciences, Haikou 571100, China; chaozhe.cn@126.com

**Keywords:** pig, meat quality, *Prox1*, variation, association analysis

## Abstract

**Simple Summary:**

The improvement of porcine meat quality is one of the most challenging tasks in pig breeding. The identification and utilization of major genes and variations affecting meat quality traits has become a research hotspot in molecular breeding of pigs. In this study, we analyzed the characteristics of porcine *Prospero-related homeobox 1* (*Prox1*) gene to lay a solid foundation for further functional study. The expression pattern of *Prox1*, as well as the correlation and association analysis of *Prox1* with meat quality traits, indicates that *Prox1* can be targeted to improve porcine meat quality traits.

**Abstract:**

*Prox1* is involved in muscle fiber conversion, adult-onset obesity, and type 2 diabetes. However, information regarding porcine *Prox1* and its relationship with meat quality traits is still unknown. In this study, we characterized the full-length cDNA and proximal promoter of two transcript variants of porcine *Prox1*. Moreover, *Prox1* was expressed abundantly in the skeletal muscle and its expression was higher in the soleus muscle than that in the biceps femoris muscle. Its expression pattern in the high and low meat color (redness) value a* groups was similar to that of *myoglobin* and *MyHC I*, but opposed to that of *MyHC IIB*. Importantly, there was a significant positive correlation between *Prox1* expression and meat color (redness) value a* (r = 0.3845, *p* = 0.0394), and a significant negative correlation between *Prox1* expression and drip loss (r = −0.4204, *p* = 0.0232), as well as the ratio of *MyHC IIB* to *MyHC I* expression (r = −0.3871, *p* = 0.0380). In addition, we found that the polymorphisms of three closely linked SNPs in *Prox1* promoter 1 were significantly associated with pH_24h_ in a pig population. Taken together, our data provide valuable insights into the characteristics of porcine *Prox1* and indicate that *Prox1* is a promising candidate gene affecting meat quality traits.

## 1. Introduction

*Prospero-related homeobox 1* (*Prox1*) encodes a homeobox transcription factor in vertebrates. Previous studies have demonstrated that *Prox1* is essential for the embryonic development of the liver [1], lymphatic system organs [2,3], pancreas [4], lens [5], and retina [6]. Moreover, adult-onset obesity caused by lymphatic vascular defects was observed in *Prox1* haploinsufficient mice [2]. *Prox1* also plays an important role in regulating hepatic triglycerides levels by mediating the HDAC3-HNF4α pathway [7], indicating that *Prox1* is critical for adipogenesis and fat storage. Recent studies have shown that *Prox1* plays a pivotal role in heart development [8] and skeletal muscle fiber conversion [9,10,11]. In our previous study, we detected differentially expressed genes (DEGs) between the classic red muscle (soleus muscle) and white muscle (biceps femoris muscle) with various muscle fibers, and identified *Prox1* as a major DEG [12]. These studies suggest that *Prox1* can be a potential candidate for improving growth and development of pigs, as well as their meat quality after slaughter.

Vertebrate *Prox1* is homologous to the *Drosophila* homeobox protein prospero, which was discovered in 1991 as a key determinant of cell fate in the *Drosophila* central nervous system [13]. Human *PROX1* and mouse *Prox1* were identified in 1993 [14] and 1996 [15], respectively. The homeo-prospero domain is highly conserved among vertebrates [16]. However, the complete sequence for most species has not been reported yet. While the partial predicted sequence of porcine *Prox1* is available in the National Center for Biotechnology Information (NCBI) database, the full-length cDNA and promoter sequences have not been reported yet. Furthermore, information regarding the role of *Prox1* in the growth and development of pigs is still lacking. 

To investigate the characteristics of porcine *Prox1* and its relationship with meat quality traits, we performed cloning, expression pattern, and promoter activity analysis of porcine *Prox1*. We also performed correlation analysis between *Prox1* expression and meat quality traits, and association analysis of the polymorphisms of SNPs g. −930 bp, g. −1421 bp, and g. −1573 bp in *Prox1* with meat quality traits. In this study, we cloned the full-length cDNA of two transcript variants of porcine *Prox1*, and observed that the coding DNA sequence (CDS) of porcine *Prox1* is conserved with that of *Prox1* in other vertebrates. Moreover, we found that porcine *Prox1* was abundantly expressed in the liver, heart, and skeletal muscle, and preferentially expressed in slow muscle, characterized by stronger oxidative metabolism capacity when compared with fast muscle with higher glycolytic metabolism capacity. Furthermore, we successfully cloned porcine *Prox1* proximal promoter sequences of two transcript variants and confirmed the core regulatory regions. In addition, we observed that *Prox1* expression was significantly correlated to meat color (redness) value a*, drip loss, and the ratio of *MyHC IIB* to *MyHC I* expression levels. Finally, we identified 18 variations in proximal promoter 1 of *Prox1*, and found that the polymorphisms of three closely linked SNPs were significantly associated with pH_24h_. Taken together, our data provide useful information for an in-depth study of porcine *Prox1* as a candidate gene to improve the growth and development of pigs, as well as their meat quality.

## 2. Materials and Methods

### 2.1. Ethics Committee

All procedures involving pigs were performed in compliance with the guideline for the care and use of experimental animals, established by the Ministry of Agriculture of China. All experiments were approved by the Institutional Animal Care and Use Committee of Nanjing Agricultural University (SYXK2011-0036).

### 2.2. Animals and Sample Collection

Tissues from the heart, liver, spleen, kidney, fat, and muscles were collected from three adult Duroc × Meishan pigs, as described by Li et al. [12], and tissues from the heart, liver, spleen, lung, kidney, stomach, and muscles were collected from three 70-day fetuses of a pregnant Landrace female crossed with a pure Landrace boar for expression analysis. The longissimus dorsi muscles at different developmental stages were collected from three fetuses of Landrace pregnant females crossed with a pure Landrace boar at 70 and 110 days post-conception (dpc), and from three pigs at two postnatal periods (1 and 70 days after birth). In addition, 32 longissimus dorsi muscles with different meat color (redness) values (a*) from a population of 279 commercial hybrid pigs (Pietrain (P) × Duroc (D)) × (Landrace (L) × Yorkshire (Y)) were collected to determine the relationship between *Prox1* expression and skeletal muscle fiber type. All the pigs were stunned by electricity and slaughtered humanely in a standardized commercial processing plant (Jiangsu Sushi Group, Jiangsu, China).

The 279 commercial hybrid pigs provided by Guangdong Wen’s Foodstuffs Group Co. Ltd. (Huaian, China) were also used for traits association analysis. The experimental pigs were raised under standard conditions and fed ad libitum with free access to water. Ear tissues for genetic diversity analysis were collected from 133 pigs using ear clips, belonging to seven breeds: three Western lean-type breeds (22 Landrace, 22 Yorkshire, and 9 Duroc pigs), three Chinese indigenous breeds (21 Erhualian, 20 Meishan, and 20 Mi pigs), and a cultivated breed (20 Suhuai pigs with 75% Yorkshire and 25% Huai heritage).

### 2.3. RNA Extraction 

Total RNA was extracted using TRIzol reagent (Invitrogen, Carlsbad, CA, USA) according to the manufacturer’s instructions. RNA integrity was checked by electrophoresis on a 1% agarose gel, and the RNA concentration and purity were determined using NanoDrop 2000 Spectrophotometer (Thermo Fisher Scientific Inc., Waltham, MA, USA). 

### 2.4. Cloning of Full-Length cDNA and Proximal Promoter 

The mRNA and genomic DNA from Erhualian pigs were used to clone full-length cDNA and the proximal promoter of *Prox1*. The predicted partial sequence of porcine *Prox1* mRNA (NM_001128490.1) was downloaded from the National Center for Biotechnology Information (NCBI) database. Three pairs of gene-specific primers (Table S1) were designed to amplify the partial sequence of porcine *Prox1* mRNA, while the 5′-end and 3′-end sequences were amplified using the SMARTer^®^ RACE 5′/3′ Kit (Clontech Laboratories, Inc., Mountain View, CA, USA), according to the user manual. The amplified fragments were cloned into the pMD19-T vector (TaKaRa Biotechnology, Dalian, China) and sequenced by Tsingke Company (Nanjing, China). Next, the full-length cDNA sequence was assembled using the SeqMan software (DNASTAR, INC., Madison, WI, USA). Subsequently, the predicted proximal promoter sequence of porcine *Prox1* was determined by performing a comparative analysis between the porcine *Prox1* full-length cDNA and porcine reference genome sequence (*Sscrofa10.2*). Five pairs of gene-specific primers were designed to amplify the proximal promoter sequences of porcine *Prox1*. The primer sequences used in the amplification of the porcine *Prox1* full-length cDNA and the proximal promoter sequences are shown in Table S1.

### 2.5. Sequence Analysis

The nucleotide and protein sequences for various species downloaded from the NCBI database are as follows: human *(Homo sapiens*, variant 1: NM_001270616.1 and NP_001257545.1; variant 2: NM_002763.4 and NP_002754.2), monkey (*Macaca mulatta*, NM_001260873.1 and NP_001247802.1), mouse (*Mus musculus*, variant 1: NM_008937.3 and NP_032963.1; variant 2: NM_001360827.1 and NP_001347756.1), and cattle (*Bos taurus*, NM_001193232.1 and NP_001180161.1). The open reading frames (ORFs) for the porcine *Prox1* gene were predicted using NCBI’s online ORFfinder tool (https://www.ncbi.nlm.nih.gov/orffinder/). Multiple sequence alignment was performed using the DNAMAN software. The potential binding sites of putative transcriptional factors were predicted using the online software JASPAR version 2016 (http://jaspar2016.genereg.net/).

### 2.6. Gene Expression and Correlation Analysis

The first strand cDNA was synthesized using the PrimerScript real-time polymerase chain reaction (RT-PCR) kit (TaKaRa Biotechnology, Dalian, China) for expression analysis. Quantitative real time PCR (qRT-PCR) primer pairs (Table S1) were designed using the online Primer 3 software. The gene expression pattern was determined using the AceQ qPCR SYBR Green Master Mix (Vazyme, Nanjing, China). All PCR reactions were performed in triplicate for each sample. The relative expression levels of porcine *Prox1*, *myoglobin*, *MyHC I*, *MyHC IIB*, and *MyHC IIX* were quantified using the 2^−△△ct^ value method, and normalized with the porcine housekeeping gene *HPRT*. 

### 2.7. Promoter Activity Analysis

Based on the two transcript variants of porcine *Prox1*, we constructed five luciferase reporter vectors containing truncated *Prox1* promoter fragments from promoters 1 and 2. Briefly, *Sac*I or *Kpn*I and *Hin*dIII restriction enzyme sites were added to the 5′-and 3′-end of the five pairs of gene-specific primers to amplify the proximal promoter fragments of porcine *Prox1* (Table S1). The promoter 1 fragments were inserted into the pGL3-bascic vector (Promega, Madison, WI), and the recombinant constructs were named pGL3-basic-P11 (+318/+122), pGL3-basic-P12 (+318/−192), pGL3-basic-P13 (+318/−682), pGL3-basic-P14 (+318/−1182), and pGL3-basic-P15 (+318/−1987). The promoter 2 fragments were inserted into the pGL3-bascic vector (Promega, Madison, WI), and the recombinant constructs were named pGL3-basic-P21 (+318/+122), pGL3-basic-P22 (+318/−192), pGL3-basic-P23 (+318/−682), pGL3-basic-P24 (+318/−1182), and pGL3-basic-P25 (+318/−1987). The sites of promoter fragments were relative to the transcription start site (+1) of *Prox1*. Subsequently, PK15 and 293T cells were cultured in 24-well plates and the recombinant constructs were transfected using Lipofectamine 3000 (Thermo Fisher Scientific, MA, USA). The activities of the promoter fragments were analyzed after 24 h transfection using the Dual-Luciferase^®^ Reporter Assay System (Promega, Madison, WI, USA) on a Glomax^®^ 20/20 luminometer platform (Promega, Madison, WI, USA). Each dual-luciferase reporter assay was repeated five times. Firefly luciferase activity was normalized to *Renilla* luciferase activity.

### 2.8. Identification of Variations 

Variations in the *Prox1* coding region were detected using cloning and sequencing methods. DNA samples were collected from seven breeds (three pigs from each breed), including three Western lean-type breeds (Landrace, Yorkshire, and Duroc), three Chinese indigenous breeds (Erhualian, Meishan, and Mi), and a cultivated breed (Suhuai). *Prox1* variation sites were confirmed by sequence alignment analysis using the DNAMAN software (Lynnon Biosoft, San Ramon, CA, USA). Variations in the proximal promoter 1 region were identified using targeted sequencing methods, as described previously by Wu et al. [17]. DNA samples derived from seven breeds, including three Western lean-type breeds (22 Landrace, 22 Yorkshire, and 9 Duroc), three Chinese indigenous breeds (21 Erhualian, 20 Meishan, and 20 Mi), a cultivated breed (20 Suhuai), and 66 ((P) × (D)) × ((L) × (Y)) commercial pigs were used for the amplification of target sequences. The amplification primers are also shown in Table S1. The positions of variations were defined relative to the transcription start site (+1) of porcine *Prox1*. Genotype and allelic frequencies were calculated manually. 

### 2.9. Traits and Genotyping

A total of 21 production traits in 279 commercial hybrid pigs were measured or recorded. The experimental pigs were slaughtered humanely in a standardized commercial processing plant (Jiangsu Sushi Group, Jiangsu, China) at an average age of 176 days in 11 random batches. Both left and right side carcass weights of each pig were measured together using the measurement tools in the slaughter system, and the backfat thickness of the last rib was measured using a digital Vernier caliper. The pH values were measured using an Hanna Instruments (HI) 9125 portable pH meter (Hanna, Thornleigh, NSW, Australia). Cooking loss was measured according to the international and national standard protocols. Shear force was measured using a C-LM3 digital tenderness instrument according to the manufacturer’s instructions. Meat color values lightness (L*), redness (a*), and yellowness (b*) were determined using a portable Minolta colorimeter (CR-10, Minolta, Japan). Drip loss was measured using the bag method. Intramuscular fat content (IMF) was measured using the Soxhlet extraction method. Muscle glucose (MG), glycogen (G), and lactate content were determined using the glucose, glycogen, and lactate test kits (Jiancheng, Nanjing, China), respectively. Glucose-6-phosphate (G6P) levels were determined using the Glucose-6-Phosphate Assay Kit (Sigma, Spruce Street, St. Louis, MO, USA). Glycolytic potential (GP) = 2 × ((glycogen) + (glucose) + (glucose-6-phosphate)) + (lactate). 

Based on sequencing results of the target region, we selected SNPs SNV00011 (g. −930 bp), SNV00014 (g. −1421 bp), and SNV00018 (g. −1573 bp) in the proximal promoter 1 of *Prox1*, and conducted genotyping in 279 commercial hybrid pigs, which consisted of 66 previously sequenced commercial pigs ((P) × (D)) × ((L) × (Y)), using the PCR restriction fragment length polymorphism (PCR-RFLP) method. The amplification primers for PCR-RFLP and the corresponding restriction enzymes are shown in Table S1. 

### 2.10. Statistical Analysis

The statistically significant difference for gene expression was performed using the unpaired sample *t* test and one-way analysis of variance (ANOVA) in SPSS 20.0. Here, *p* < 0.05 was considered to be statistically significant and the data are presented as the mean ± SEM (n = 3). Statistically significant differences for meat color (redness) value a* were assessed using the unpaired sample *t* test in SPSS 20.0. Here, *p* < 0.05 was considered to be statistically significant and data are presented as the mean ± SEM (n = 17 and 15). Correlation analysis between the expression of *Prox1* and *myoglobin*, *MyHC I*, *MyHC IIB*, *MyHC IIX*, and phenotype traits was assessed in SPSS 20.0. Statistically significant differences for promoter activity were assessed using ANOVA with Duncan’s multiple range tests in SPSS 20.0. Here, *p* < 0.05 was considered to be statistically significant and data are presented as the mean ± SEM (n = 5). The association between the polymorphisms of the three selected SNPs (g. −930 bp, g. −1421 bp, g. −1573 bp) and carcass and meat quality traits was assessed using the general linear model (GLM) procedure in SAS version 8.0 (SAS Institute, Cary, NC, USA). The genotype, sex, and batch were used as fixed effects in the model, with carcass weight as a covariate.

## 3. Results and Discussion 

### 3.1. Characteristics of Porcine Prox1 Sequences

We performed 5′ and 3′ rapid amplification of cDNA ends-PCR (RACE-PCR) to obtain the full-length cDNA of porcine *Prox1*. Two kinds of alternative splicing forms were found in the 5′-end sequence of *Prox1*. All *Prox1* cDNA fragments (except the 5′-end sequence fragments) were amplified and sequenced; however, only one form was identified. Therefore, we deduced that at least two putative transcript variants exist in porcine *Prox1*. The first and major variant (transcript variant 1) contained five exons and four introns, and encoded 737 amino acids. The 3683 bp full-length cDNA of transcript variant 1 (GenBank Accession No: MK704404) contained a 2214 bp ORF flanked by a 484 bp 5′-untranslated region (5′ UTR) and 985 bp 3′ UTR. The second variant (transcript variant 2) contained four exons and three introns because the transcription start site (TSS) was present at the first intron of transcript variant 1. The 4236 bp full-length cDNA of transcript variant 2 (GenBank Accession No: MK704405) contained a 1037 bp 5′ UTR and 985 bp 3′ UTR.

The transcript variant 1 was identified as the major form because the sequencing data revealed a few clones of transcript variant 2 clones in sequencing data. The two transcript variants of human *PROX1* and mouse *Prox1* are deposited in the NCBI database and the nucleotide sequences of the coding region of both transcript variants are identical. Therefore, the coding region sequence of the porcine transcript variant 2 may be similar to transcript variant 1. Moreover, we performed multiple sequence alignment of the *Prox1* coding region nucleotide sequences, as well as protein sequences for different species, including human, mouse, monkey, and cattle. The coding region nucleotide sequences showed 96.58% identity among all species, while the protein sequences were 99.19% identical (Figure S1 and Figure S2). These indicate that *Prox1* is highly conserved among vertebrates, and might exhibit similar function in different species. In addition, we performed transcript factor prediction for *Prox1* promoter 1 using the online software JASPAR (http://jaspar.genereg.net). A total of 434 non-redundant potential transcript factors were found in porcine *Prox1* promoter 1 (Table S2).

### 3.2. Expression Patterns of Porcine Prox1

**Figure 1:**
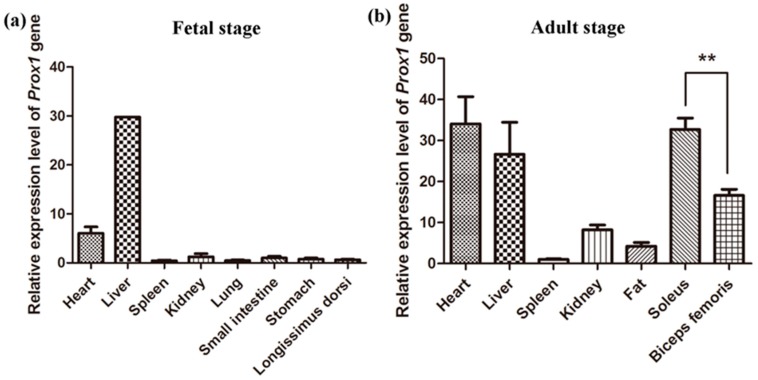
Expression pattern of porcine *Prox1*. (**a**) Expression pattern of porcine *Prox1* in fetal tissues. (**b**) Expression pattern of porcine *Prox1* in adult tissues. Real-time polymerase chain reaction (PCR) was used to determine the expression levels of *Prox1* genes. All PCR reactions were performed in triplicate for each sample. Relative expression levels were calculated using the 2^−△△ct^ value method and normalized with the porcine housekeeping gene *HPRT*. Data are presented as the mean ± Standard Error of Mean (SEM) (n = 3). Statistical analysis was performed using the unpaired sample *t* test in SPSS 20.0. Note: ** indicates *p* < 0.01.

We determined the expression patterns of *Prox1* in various tissues of adult pigs and 70-day fetuses using real-time PCR. Our results showed that porcine *Prox1* is abundantly expressed in the heart and liver of both adult pigs and fetuses. However, *Prox1* expression is low in the lung, spleen, kidney, stomach, and fat tissues (Figure 1a,b). 

**Figure 2:**
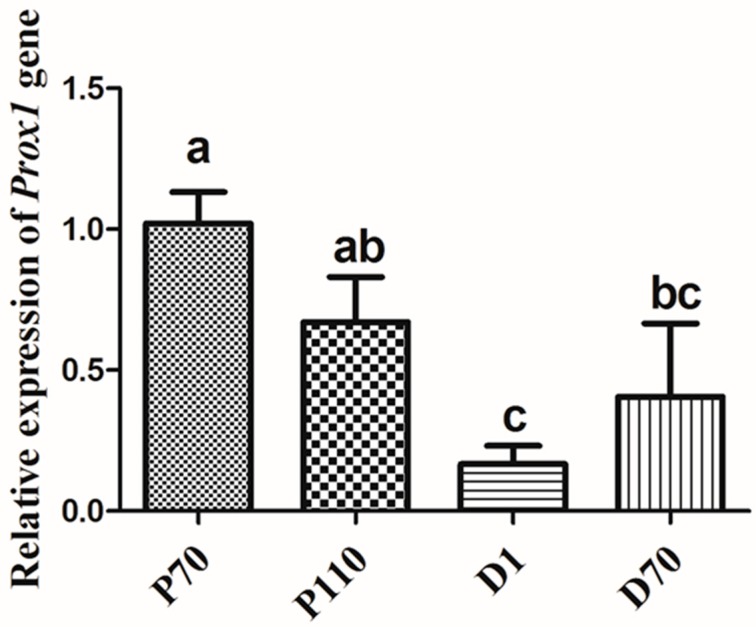
Expression pattern of porcine *Prox1* at different developmental stages. Longissimus dorsi muscles were collected from three fetuses of Landrace pregnant females at 70 days (P70) and 110 days (P110) post conception (dpc), and from three pigs on day 1 (D1) and at 70 days (D70) after birth. Real-time PCR was performed to determine *Prox1* expression levels. All PCR reactions were performed in triplicate for each sample. The relative expression levels were calculated using the 2^−△△ct^ value method and normalized with porcine housekeeping gene *HPRT*. Statistical analysis was performed using one-way ANOVA with Duncan’s multiple range tests. Data are presented as the mean ± SEM (n = 3). Different letters above the bars indicate significant differences. Note: *p* < 0.05.

The high expression of porcine *Prox1* in the liver and heart of adult pigs and fetuses suggests its importance in the development of embryonic heart [8] and liver [1]. *Prox1* also has been shown to play a role in the development of the murine lymphatic system [2,3], retina [5,6], pancreas [4], and skeletal muscle [9]. Notably, *Prox1* expression was lower in skeletal muscle when compared with that in the heart and liver at the fetal stage, but was highly expressed in skeletal muscle as well as heart and liver at the adult stage (Figure 1b), suggesting that *Prox1* is critical for skeletal muscle, heart, and liver development in adult pigs. In humans, *PROX1* is involved in the hepatic maturation of human-induced pluripotent stem-cell-derived hepatocyte-like cells (iPS-HLCs) and can promote hepatocellular carcinoma progression via the Wnt/β-catenin pathway [18,19]. Moreover, *Prox1* was reported to play a critical role in heart growth and development [8], which is consistent with its high expression in porcine heart tissue. We also determined porcine *Prox1* expression in the skeletal muscle at different developmental stages before and after birth. *Prox1* expression was relatively higher before birth (Figure 2), although the possibility of its increased expression is not excluded at later developmental stages. 

Several recent studies have indicated that *Prox1* is essential for skeletal muscle growth and development, and can regulate muscle fiber conversion by interacting with the nuclear factor of activated T cells (NFAT) and Notch pathways [9,11]. Our results showed that porcine *Prox1* expression in the soleus muscle (red muscle) is higher than that in the biceps femoris muscle (white muscle) (Figure 1b), which also suggests that porcine *Prox1* plays an important role in skeletal muscle fiber conversion. Moreover, *Prox1* is preferentially expressed in slow muscle, characterized by stronger oxidative metabolism capacity in zebrafish [20] and mice [10]. In addition, *Prox1* expression is significantly increased in fast muscle gene *Sox6* knockout muscles, suggesting that *Prox1* is critical for the conversion of slow to fast muscle fibers [10]. 

**Figure 3:**
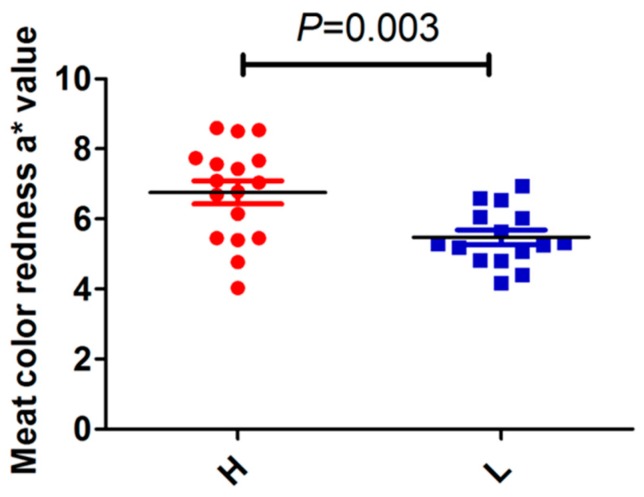
Comparison of meat color (redness) value a* between high and low groups. Thirty-two longissimus dorsi muscles were divided into two groups according the ratio of *MyHC IIB* to *MyHC I* expression (threshold value = 2.0). The ratios of > 2.0 and < 2.0 were defined as high meat color a* group (H) and low meat color a* group (L), respectively. Statistical analysis between the H and L groups was performed using the unpaired sample *t* test method in SPSS 20.0. Data are presented as the mean ± SEM (n = 17 for the H group and n = 15 for the L group).

To demonstrate the relationship between porcine *Prox1* expression and skeletal muscle fiber type, we randomly selected 32 longissimus dorsi muscles from a population of 279 commercial hybrid pigs ((P) ×(D)) × ((L) × (Y)) and determined the expression patterns of porcine *Prox1*, *myoglobin*, *MyHC I*, *MyHC IIB*, and *MyHC IIX*. The samples were divided into two groups according the ratio of *MyHC IIB* to *MyHC I* expression level; a ratio of > 2.0 was defined as high meat color a* group (H) and a ratio < 2.0 was defined as low meat color a* group (L). Our results indicated that a ratio of 2.0 can be used as the threshold value to distinguish the meat color (redness) value (a*) measured 24 h after slaughter (Figure 3), which also suggests that it is possible to predict meat color (redness) using the expression pattern of porcine *Prox1*. 

**Figure 4:**
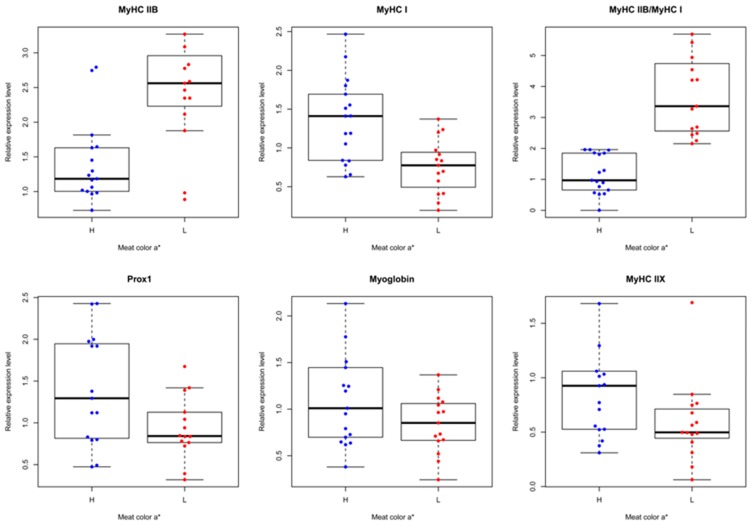
Comparison of porcine *Prox1* expression to that of skeletal muscle-fiber-related genes. Thirty-two longissimus dorsi muscles from a population of 279 commercial hybrid pigs were randomly selected, and the expression patterns of porcine *Prox1* and skeletal muscle-fiber-related genes (including *myoglobin*, *MyHC I*, *MyHC IIB*, and *MyHC IIX*) were determined using real-time PCR. All PCR reactions were performed in triplicate for each sample. The relative expression levels were calculated using the 2^−△△ct^ value method and normalized with the porcine housekeeping gene *HPRT*. The samples were divided into two groups according the ratio of *MyHC IIB* to *MyHC I* expression level, which was set at 2.0.

Our results showed that the expression pattern of porcine *Prox1* in the H and L groups was similar to that of *myoglobin* and *MyHC I*, but opposed to that of *MyHC IIB* (Figure 4), indicating that porcine *Prox1* may be closely related to skeletal muscle fiber diversity. These results further confirm the importance of *Prox1* in skeletal muscle fiber conversion [9,11].

### 3.3. Correlation Analysis of Prox1 Expression with Meat Quality Traits

**Figure 5:**
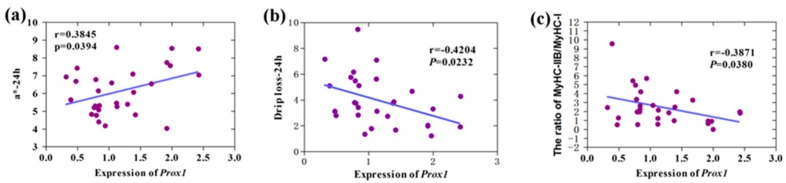
Correlation between porcine *Prox1* expression and meat quality traits. The expression of porcine *Prox1* was compared with that of *myoglobin*, *MyHC I*, *MyHC IIB*, and *MyHC IIX* in Figure 4, and the phenotype data of 32 commercial hybrid pigs are shown in Table 2. (**a**) Correlation between *Prox1* expression and meat color (redness) value a* at 24 h postmortem. (**b**) Correlation between *Prox1* expression and drip loss at 24 h postmortem. (**c**) Correlation between *Prox1* expression and the ratio of *MyHC IIB* to *MyHC I* expression levels.

Skeletal muscles composed of different fibers exhibit different physiological and metabolic properties, including strength, twitch speed, and endurance, and oxidative and glycolytic capacity [21]. After slaughter, muscles composed of different fibers are subjected to various changes during the conversion of muscle to meat. Therefore, the type of skeletal muscle fiber definitely affects meat quality traits, such as pH, meat color, and drip loss [22,23]. Our results showed that *Prox1* expression is significantly positively correlated to meat color (redness) value a* at 24 h postmortem (r = 0.3845, *p* = 0.0394), but significantly negatively correlated to drip loss at 24 h postmortem (r = −0.4204, *p* = 0.0232) (Figure 5). 

A significant correlation was also observed between *Prox1* expression and the ratio of *MyHC IIB* to *MyHC I* expression level (r = −0.3871, *p* = 0.0380). It is known that skeletal muscles with different muscle fibers, such as biceps femoris and soleus muscles in the pigs, show obvious differences in the meat color [12], and exhibit different glycolytic capacities, accounting for muscle pH decline postmortem. Moreover, the change of pH is closely related to drip loss. Notably, the correlation analysis of meat quality traits in the population of 279 commercial hybrid pigs used in this study indicated that meat color is significantly related to drip loss. Therefore, our data suggest that *Prox1* improves the meat quality by regulating skeletal muscle fiber conversion. 

### 3.4. Identification of Core Promoter Region

**Figure 6:**
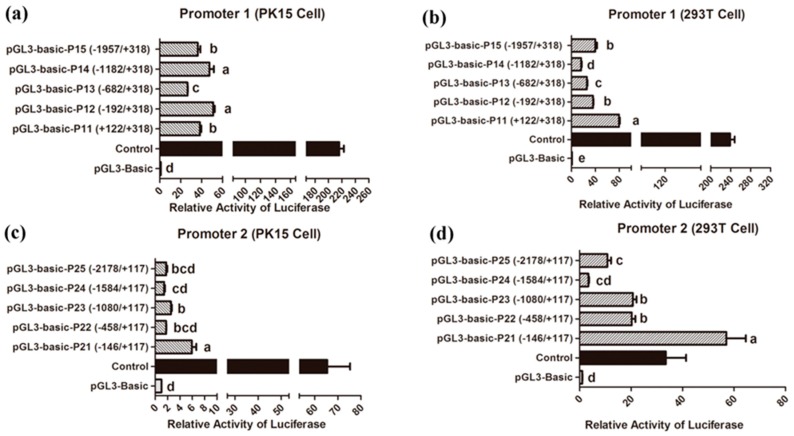
Promoter activity analysis for the two transcript variants of porcine *Prox1*. Promoter activity analysis for *Prox1* transcript variant 1 (Promoter 1) in PK15 cells (**a**) and 293T cells (**b**). Promoter activity analysis for *Prox1* transcript variant 2 (Promoter 2) in PK15 cells (**c**) and 293T cells (**d**). The left side of the x-axis represents a series of truncated 5′-flanking sequences of *Prox1* that were fused to the firefly luciferase reporter gene in the pGL3-basic vector, and their positions were defined relative to the transcription start site (+1) of porcine *Prox1*. The right side of the x-axis indicates the relative luciferase activity of promoter fragments. Here, pGL3-basic and -control were the negative and positive control vectors, respectively. The pRL-TK vector encoding *Renilla* luciferase was co-transfected as the internal reference. Promoter activity was defined by the normalization of firefly luciferase activity to *Renilla* luciferase activity, and the value of pGL3-basic negative vector was defined as 1. Data are presented as the mean ± SEM (n = 5). Statistical analysis was performed in SPSS 20.0 using one-way ANOVA test with Duncan’s multiple range tests. Data are presented as the mean ± SEM (n = 5). Different letters above the bars indicate significant differences. Note: *p* < 0.05.

We cloned the two proximal promoters of the porcine *Prox1* transcript variants and identified the promoter active region using the Dual-Luciferase^®^ Reporter Assay System. The promoter activity was analyzed in PK15 and 293T cells. As shown in Figure 6a,b, the luciferase activity of all promoter 1 fragments was significantly higher than that of the pGL3-basic negative control, although the promoter activity differed among the different fragments in PK15 and 293T cells. The most active regions of promoter 1 were located between +318 bp and −192 bp (pGL3-basic-P11) in PK15 cells and between +318 bp and +122 bp (pGL3-basic-P11) in 293T cells. Moreover, based on the luciferase activity results of different promoter fragments in 293T cells, it can be deduced that some negative elements may exist in the regions from −1182 to −682 bp, from −682 to −192 bp, and from −192 to +122 bp. However, the region from −1957 to −1182 bp may contain potential positive elements. Similarly, negative elements may exist in the region from −682 to −192bp in PK15 cells. These results suggest that the regulatory mechanisms of promoter 1 may vary among different cells. In addition, the luciferase activity of all promoter 2 fragments showed that there were large differences between the 293T cells and PK15 cells; the promoter activity in the 293T cells was stronger than that in the PK15 cells (Figure 6c,d). Of note, the most active region of promoter 2 was consistently located between −146 bp and +117 bp (pGL3-basic-P21), both in 293T and PK15 cells. However, differences in the luciferase activity were still observed, suggesting that it is a core regulatory region for promoter 2 activity.

### 3.5. Genetic Diversity of Sequence Variations

No variation was detected in the coding region of porcine *Prox1*. However, 18 variations in the *Prox1* proximal promoter 1 region were identified using targeted sequencing methods. The detailed information regarding each variation, including the location of gene body, variation type, and reference genome position, are shown in Table 1. 

To understand the genetic diversity of these variations, we conducted genotype and allele frequency analyses in three Western lean-type breeds (Landrace, Yorkshire, and Duroc), three Chinese indigenous breeds (Erhualian, Meishan, and Mi), a cultivated breed (Suhuai), and 66 commercial pigs ((P) × (D)) × ((L) × (Y)). The genotypes and allele frequencies of all variations are shown in Table S3. We found that SNV00003, SNV00005, SNV00009, SNV00010, SNV00011, SNV00014, SNV00015, and SNV00018 variations showed extensive polymorphisms among different pig breeds. Notably, we found the SNV00010 and SNV00011 variations are completely linked in all tested pig breeds. In humans, several SNPs in *Prox1* have been shown to significantly correlate with type 2 diabetes, and the underling mechanism is thought to be mediated by affecting glucose or lipid metabolism [24,25,26,27]. However, the correlation between the polymorphisms of specific SNPs in porcine *Prox1* and porcine production traits, especially growth, carcass, and meat quality traits, remain unclear. Hence, we subsequently performed trait association analysis.

### 3.6. Trait Association Analysis

We found the polymorphisms of three variations, namely, SNV00011 (g. −930 bp), SNV00014 (g. −1421 bp), and SNV00018 (g. −1573 bp), were obviously different among the Western lean-type and Chinese indigenous breeds, and they were highly linked. We further performed genotyping in the 279 commercial hybrid pigs using the PCR-RFLP method and a trait association analysis. The genotypic gel electrophoresis results are shown in Figure S3. The trait phenotype with the mean values, SD, and coefficient of variation (CV) are shown in Table 2. 

The data showed that the coefficient of variation (CV) of backfat, intramuscular fat content (IMF), color a*, color b*, drip loss, shear force, and muscle glycogen exceeded 20%, indicating a large variation in phenotypic trait values existed, which provided a good basis for trait association analysis in the ((P) × (D)) × ((L) × (Y)) commercial pig population. The genotype results (Table S3) showed that three SNPs are highly linked in the ((P) × (D)) × ((L) × (Y)) commercial pig population. As the number with AA genotype at g. −930 bp site, GG genotype at g. −1421 bp site, and GG genotype at g. −1573 bp site was limited (n = 8), we performed an association analysis for the pigs with the other two genotypes at each SNP. The results (Table 3) indicated that the polymorphisms of the three SNPs are significantly associated with pH_24h_ (*p* = 0.022). Th results indicate that pH decline is the major factor causing drip loss, and pH value will be stable at 24 h or longer. So, drip loss measured at 24 h and 48 h was commonly used for meat quality assessment after slaughter. Notably, the DL_24h_ among various genotypes showed suggesting differences (*p* = 0.070), and the homozygous genotype pigs with higher pH_24h_ correspondingly showed lower DL_24h_ and DL_48h_, suggesting that allele C at g. −930 bp, allele A at g. −1421 bp, and allele C at g. −1573 bp are favorable alleles for better meat quality.

The pH value is a critical parameter for evaluating meat quality. Low pH denatures muscle proteins and reduces protein solubility, thereby affecting meat quality [28,29,30,31,32]. For example, pale, soft, and exudative (PSE) meat is produced by a rapid decrease in the pH value after slaughter, and dark, firm, and dry (DFD) meat is generated by a high ultimate pH value [33]. The pH value is directly influenced by glycolysis, in which glycogen is converted to lactate and H^+^. Recent studies have shown that *Prox1* is significantly correlated with type 2 diabetes, and the underlying mechanism is mediated by affecting glucose metabolism [24,25,26,27,34,35]. Importantly, *Prox1* was demonstrated to regulate the muscle fiber conversion, and the loss of *Prox1* promotes conversion of slow to fast muscle fibers [9,20]. Skeletal muscles with various muscle fibers display different properties; slow muscle fibers (type I and type IIa) exhibit high oxidative metabolism capacity, while fast muscle fibers (type IIx and type IIb) exhibit high glycolytic metabolism capacity [21,36]. Therefore, the association of porcine *Prox1* with pork pH value may be mediated by the regulation of skeletal muscle fiber type and glucose metabolism. Moreover, pH value is closely related to other meat quality traits, such as drip loss [37,38]. Our results obtained from the phenotype data in pig populations used in this study also indicated that pH_45min_ and pH_24h_ both exhibit a strong negative effect on DL_24h_ and DL_48h_ (data not shown), which is consistent with the three SNPs polymorphisms of porcine *Prox1*, suggesting association with DL_24h_. Generally, these results are consistent with the conclusion that the expression of *Prox1* is significantly correlated to meat color (redness) value a* and drip loss at 24 h postmortem (Figure 5).

## 4. Conclusions

*Prox1* is an essential gene for skeletal muscle development and muscle fiber conversion. Porcine *Prox1* is a significantly DEG between the skeletal muscles with different muscle fibers. The present study firstly analyzed the characteristics of porcine *Prox1*, laying a solid foundation for future functional studies. The expression pattern of *Prox1*, as well as the correlation and association analysis of *Prox1* with meat quality traits, indicates that *Prox1* can be targeted to improve porcine meat quality traits. Overall, our data provide a basis for the application of *Prox1* gene in pig genetics to improve meat quality traits.

## Figures and Tables

**Table 1:** Variations identified in the porcine *Prox1* promoter 1.

Gene Localization	Polymorphism	Chromosome	Reference Genome Position ^1^
g. +123	G/A	9	142477946
g. −268	T/G	9	142478335
g. −414	C/T	9	142478482
g. −427	C/A	9	142478495
g. −502	G/A	9	142478570
g. −516	A/C	9	142478584
g. −759	C/T	9	142478827
g. −780	AGA/-	9	142478846-142478848
g. −848	-/A	9	142478913-142478913
g. −924	T/C	9	142478992
g. −930	C/A	9	142478998
g. −1143	G/T	9	142479211
g. −1179	C/G	9	142479247
g. −1421	A/G	9	142479489
g. −1448	AC/-	9	142479515-142479516
g. −1452	ACACAC/-	9	142479515-142479520
g. −1454	ACACACAC/-	9	142479515-142479522
g. −1573	C/G	9	142479641

^1^*Sus scrofa* 10.2.

**Table 2:** Phenotype information for experimental pig population.

Trait ^1^	N ^2^	Mean ± SD ^3^	CV% ^4^
Age (d)	279	176.92 ± 4.14	2.34
CW (kg)	279	82.50 ± 11.10	13.46
BF (mm)	279	1.77 ± 0.48	27.13
pH_45min_	279	6.22 ± 0.30	4.78
pH_24h_	279	5.58 ± 0.18	3.28
IMF (%)	279	2.64 ± 0.91	34.55
L*_45min_	129	45.37 ± 2.29	5.04
a*_45min_	129	3.85 ± 1.500	38.92
b*_45min_	129	7.67 ± 0.93	12.14
L* _24h_	279	51.49 ± 3.00	5.81
a*_24h_	279	6.32 ± 1.15	18.14
b*_24h_	279	5.88 ± 1.26	21.42
DL_24h_ (%)	279	1.75 ± 0.97	55.31
DL_48h_ (%)	279	3.89 ± 1.54	39.66
CL (%)	279	30.88 ± 3.48	11.26
SF (N)	279	47.23 ± 12.58	26.65
MG (μmol/g)	279	5.28 ± 1.71	32.44
G (μmol/g)	279	3.33 ± 0.84	25.23
G6P (μmol/g)	279	1.48 ± 0.19	12.69
LA (μmol/g)	279	130.82 ± 23.41	17.90
GP (μmol/g)	279	150.03 ± 26.27	17.50

^1^ Traits: Age, days after slaughter; CW, carcass weight; BF, backfat; pH_45min_, pH value at 45 min postmortem; pH_24h_, pH value at 24 h postmortem; IMF, intramuscular fat content; L*, lightness at 45 min or 24 h postmortem; a*, redness at 45 min or 24 h postmortem; b*, yellowness at 45 min or 24 h postmortem; DL_24h_, drip loss measured at 24 h postmortem; DL_48h_, drip loss measured at 48 h postmortem; CL, cooking loss; SF, shear force; MG, muscle glycogen; G, glucose; G6P, glucose-6-phosphate; LA, lactic acid; GP, glycolytic potential. ^2^ N = number of pigs. ^3^ SD = standard deviation. ^4^ CV = coefficient of variation.

**Table 3:** Association analysis of SNPs polymorphisms with porcine production traits.

Traits	Genotype (g. −930 bp)	Genotype (g. −1421 bp)	Genotype (g. −1573 bp)	Number	Least Squares Means (LSM) ± SE	*p* Value
pH_24h_	CA	AG	CG	87	5.55 ± 0.02	0.022
	CC	AA	CC	184	5.60 ± 0.01	
Drip loss_24h_ (%)	CA	AG	CG	87	1.95 ± 0.12	0.070
	CC	AA	CC	184	1.71 ± 0.09	
Drip loss_48h_ (%)	CA	AG	CG	87	5.04 ± 0.33	0.193
	CC	AA	CC	184	4.78 ± 0.31	

## Supplementary Materials

The following are available online at https://www.mdpi.com/2076-2615/9/10/744/s1. The sequences of the two transcript variants of porcine *Prox1* were deposited to the NCBI GenBank database under accession numbers: MK704404 and MK704405. Table S1: Primers used in this study. Table S2: Prediction of transcript factors in porcine *Prox1* promoter 1. Table S3: Genotypes and allele frequencies of variations in different pig breeds. Figure S1: Alignment of *Prox1* CDS nucleotide sequences derived from different species. Figure S2: Alignment of *Prox1* protein sequences of CDS derived from different species. Figure S3: Genotypic gel electrophoresis pattern using the PCR-RFLP method.
