# Supplementary material for: Comprehensive Analysis of Porcine Prox1 Gene and Its Relationship with Meat Quality Traits
Source: Animals (Basel). 2019 Sep 29;9(10):744. doi: 10.3390/ani9100744 (PMC6826434; doi:10.3390/ani9100744)
Supplement: Supplementary file 1 [file animals-09-00744-s001.zip › Supplementary Files/Supplementary caption.docx]

**Supplementary caption**

**Figure S1.** Alignment of *Prox1* CDS nuclotide sequences derived from different species. The nucleotide sequences of different species were obtained from NCBI database, including Human (Homo sapies, variant 1: NM_001270616.1; variant 2: NM_002763.4), Monkey (Macaca mulatta, NM_001260873.1), Mouse (Mus musculus, variant 1: NM_008937.3; variant 2: NM_001360827.1), and Cattle (Bos taurus, NM_001193232.1). Multiple sequence alignment was performed using DNAMAN software and the black background represents complete identical nucleotide.

**Figure S2.** Alignment of *Prox1* protein sequences of CDS derived from different species. The protein sequences of different species were downloaded from NCBI database, including Human (Homo sapies, variant 1: NP_001257545.1; variant 2: NP_002754.2), Monkey (Macaca mulatta, NP_001247802.1), Mouse (Mus musculus, variant 1: NP_032963.1; variant 2: NP_001347756.1), and Cattle (Bos taurus, NP_001180161.1). Multiple sequence alignment was performed using DNAMAN software and the black background represents complete identical amino acid residues.

**Figure S3.** Genotypic gel electrophotresis pattern using PCR-RFLP method. PCR-RFLP analysis of porcine *Prox1* g.-930 bp (**a**), g.-1421 bp (**b**), and g.-1573 bp (**c**) polymorphisms. 1.5% agarose gen electrophoresis was used for genotyping of g.-930 bp, g.-1421 bp and the PCR fragment for g.-930 bp and g.-1421 bp markers were digested with *Taq*I and *Bse*NI, respectively. 2% agarose gen electrophoresis was used for genotyping of g.-1573 bp and the PCR fragment was digested with *Hpa*II. The genotypes were indicated on the bottom of each laness. M, DL2000.

**Table S1.** Primers used in this study.

**Table S2.** Prediction of transcript factors in porcine *Prox1* promoter 1.

**Table S3.** Genotypes and allele frequencies of variations in different pig breeds.
