# Supplementary material for: Comprehensive Analysis of Porcine Prox1 Gene and Its Relationship with Meat Quality Traits
Source: Animals (Basel). 2019 Sep 29;9(10):744. doi: 10.3390/ani9100744 (PMC6826434; doi:10.3390/ani9100744)
Supplement: Supplementary file 1 [file animals-09-00744-s001.zip › Supplementary Files/Table S3.docx]

Characteristics of Porcine *Prox1* Gene and Its Relationship with Production Traits

Chao Dong ^1, a^, Yingxi Zhang ^1, a^, Kaiqing Liu ^1^, Bojiang Li ^1, 2^, Zhe Chao ^3^, Aiwen Jiang ^1^, Rongyang Li ^1^, Zengkai Zhang ^1^, Pinghua Li ^1^, Honglin Liu ^1^ and Wangjun Wu ^1 +^

**Supplementary Table S3 Genotypes and allele frequencies of variations in different pig breeds.**

| **Variant ID : SNV00001**  **g. +123 G/A** | | **Genotype** | | | | | | | | Allele frequency | | | | | |
| --- | --- | --- | --- | --- | --- | --- | --- | --- | --- | --- | --- | --- | --- | --- | --- |
| **Breed** | **Number** | **G/G** | **G/A** | | | A/A | | | | G | | | | | A |
| Landrace | 22 | 22 | 0 | | | 0 | | | | 1 | | | | | 0 |
| Yorkshire | 22 | 22 | 0 | | | 0 | | | | 1 | | | | | 0 |
| Duroc | 7 | 3 | 4 | | | 0 | | | | 0.714 | | | | | 0.286 |
| Suhuai | 21 | 21 | 0 | | | 0 | | | | 1 | | | | | 0 |
| Erhualian | 21 | 21 | 0 | | | 0 | | | | 1 | | | | | 0 |
| Mi | 20 | 20 | 0 | | | 0 | | | | 1 | | | | | 0 |
| Meishan | 19 | 19 | 0 | | | 0 | | | | 1 | | | | | 0 |
| PDLY | 66 | 55 | 11 | | | 0 | | | | 0.917 | | | | | 0.083 |
| Variant ID :SNV00002  g.-268 T/G | | Genotype | | | | | | | | Allele frequency | | | | | |
| Breed | Number | T/T | T/G | | | G/G | | | | T | | | | | G |
| Landrace | 15 | 15 | 0 | | | 0 | | | | 1 | | | | | 0 |
| Yorkshire | 18 | 18 | 0 | | | 0 | | | | 1 | | | | | 0 |
| Duroc | 6 | 6 | 0 | | | 0 | | | | 1 | | | | | 0 |
| Suhuai | 16 | 13 | 3 | | | 0 | | | | 0.90625 | | | | | 0.09375 |
| Erhualian | 19 | 19 | 0 | | | 0 | | | | 1 | | | | | 0 |
| Mi | 12 | 12 | 0 | | | 0 | | | | 1 | | | | | 0 |
| Meishan | 9 | 9 | 0 | | | 0 | | | | 1 | | | | | 0 |
| PDLY | 55 | 53 | 2 | | | 0 | | | | 0.9818 | | | | | 0.0182 |
| Variant ID : SNV00003  g.-414 C/T | | Genotype | | | | | | | | Allele frequency | | | | | |
| Breed | Number | C/C | C/T | | | T/T | | | | C | | | | | T |
| Landrace | 22 | 12 | 9 | | | 1 | | | | 0.75 | | | | | 0.25 |
| Yorkshire | 22 | 20 | 1 | | | 1 | | | | 0.9318 | | | | | 0.0682 |
| Duroc | 7 | 7 | 0 | | | 0 | | | | 1 | | | | | 0 |
| Suhuai | 21 | 20 | 0 | | | 1 | | | | 0.9524 | | | | | 0.0476 |
| Erhualian | 21 | 10 | 11 | | | 0 | | | | 0.7381 | | | | | 0.2619 |
| Mi | 20 | 12 | 8 | | | 0 | | | | 0.8 | | | | | 0.2 |
| Meishan | 16 | 1 | 2 | | | 13 | | | | 0.125 | | | | | 0.875 |
| PDLY | 66 | 49 | 16 | | | 1 | | | | 0.8636 | | | | | 0.1364 |
| Variant ID :SNV00004  g.-427 C/A | | Genotype | | | | | | | | Allele frequency | | | | | |
| Breed | Number | T/T | T/C | | | C/C | | | | T | | | | | C |
| Landrace | 22 | 22 | 0 | | | 0 | | | | 1 | | | | | 0 |
| Yorkshire | 22 | 22 | 0 | | | 0 | | | | 1 | | | | | 0 |
| Duroc | 7 | 7 | 0 | | | 0 | | | | 1 | | | | | 0 |
| Suhuai | 21 | 21 | 0 | | | 0 | | | | 1 | | | | | 0 |
| Erhualian | 21 | 13 | 8 | | | 0 | | | | 0.8095 | | | | | 0.1905 |
| Mi | 20 | 14 | 6 | | | 0 | | | | 0.85 | | | | | 0.15 |
| Meishan | 16 | 16 | 0 | | | 0 | | | | 1 | | | | | 0 |
| PDLY | 66 | 66 | 0 | | | 0 | | | | 1 | | | | | 0 |
| Variant ID :SNV00005  g.-502 G/A | | Genotype | | | | | | | | Allele frequency | | | | | |
| Breed | Number | G/G | G/A | | | A/A | | | | G | | | | | A |
| Landrace | 22 | 20 | 2 | | | 0 | | | | 0.9545 | | | | | 0.0455 |
| Yorkshire | 21 | 20 | 1 | | | 0 | | | | 0.9762 | | | | | 0.0238 |
| Duroc | 7 | 7 | 0 | | | 0 | | | | 1 | | | | | 0 |
| Suhuai | 21 | 21 | 0 | | | 0 | | | | 1 | | | | | 0 |
| Erhualian | 21 | 0 | 11 | | | 10 | | | | 0.2619 | | | | | 0.7381 |
| Mi | 18 | 0 | 9 | | | 9 | | | | 0.25 | | | | | 0.75 |
| Meishan | 2 | 2 | 0 | | | 0 | | | | 1 | | | | | 0 |
| PDLY | 66 | 66 | 0 | | | 0 | | | | 1 | | | | | 0 |
| Variant ID : SNV00006  g.-516 A/C | | Genotype | | | | | | | | Allele frequency | | | | | |
| Breed | Number | A/A | A/C | | | C/C | | | | A | | | | | C |
| Landrace | 22 | 22 | 0 | | | 0 | | | | 1 | | | | | 0 |
| Yorkshire | 21 | 21 | 0 | | | 0 | | | | 1 | | | | | 0 |
| Duroc | 7 | 7 | 0 | | | 0 | | | | 1 | | | | | 0 |
| Suhuai | 21 | 21 | 0 | | | 0 | | | | 1 | | | | | 0 |
| Erhualian | 21 | 21 | 0 | | | 0 | | | | 1 | | | | | 0 |
| Mi | 19 | 19 | 0 | | | 0 | | | | 1 | | | | | 0 |
| Meishan | 2 | 2 | 0 | | | 0 | | | | 1 | | | | | 0 |
| PDLY | 66 | 62 | 4 | | | 0 | | | | 0.9697 | | | | | 0.0303 |
| Variant ID :SNV00007  g.-759 C/T | | Genotype | | | | | | | | Allele frequency | | | | | |
| Breed | Number | C/C | C/T | | | T/T | | | | C | | | | | T |
| Landrace | 22 | 22 | 0 | | | 0 | | | | 1 | | | | | 0 |
| Yorkshire | 18 | 18 | 0 | | | 0 | | | | 1 | | | | | 0 |
| Duroc | 7 | 7 | 0 | | | 0 | | | | 1 | | | | | 0 |
| Suhuai | 21 | 21 | 0 | | | 0 | | | | 1 | | | | | 0 |
| Erhualian | 21 | 21 | 0 | | | 0 | | | | 1 | | | | | 0 |
| Mi | 20 | 20 | 0 | | | 0 | | | | 1 | | | | | 0 |
| Meishan | 19 | 19 | 0 | | | 0 | | | | 1 | | | | | 0 |
| PDLY | 53 | 52 | 1 | | | 0 | | | | 0.9906 | | | | | 0.0094 |
| Variant ID :SNV00008  g.-780AGA/- | | Genotype | | | | | | | | Allele frequency | | | | | |
| Breed | Number | AGA/AGA | AGA/- | | | -/- | | | | AGA | | | | | - |
| Landrace | 22 | 21 | 1 | | | 0 | | | | 0.9773 | | | | | 0.0227 |
| Yorkshire | 18 | 18 | 0 | | | 0 | | | | 1 | | | | | 0 |
| Duroc | 7 | 5 | 2 | | | 0 | | | | 0.8571 | | | | | 0.1429 |
| Suhuai | 21 | 20 | 1 | | | 0 | | | | 0.9762 | | | | | 0.0238 |
| Erhualian | 21 | 21 | 0 | | | 0 | | | | 1 | | | | | 0 |
| Mi | 20 | 20 | 0 | | | 0 | | | | 1 | | | | | 0 |
| Meishan | 19 | 19 | 0 | | | 0 | | | | 1 | | | | | 0 |
| PDLY | 53 | 49 | 4 | | | 0 | | | | 0.9623 | | | | | 0.0377 |
| Variant ID : SNV00009  g.-848 -/A | | Genotype | | | | | | | | Allele frequency | | | | | |
| Breed | Number | -/- | -/A | | | A/A | | | | - | | | | | A |
| Landrace | 22 | 6 | 16 | | | 0 | | | | 0.6364 | | | | | 0.3636 |
| Yorkshire | 17 | 15 | 2 | | | 0 | | | | 0.9412 | | | | | 0.0588 |
| Duroc | 7 | 7 | 0 | | | 0 | | | | 1 | | | | | 0 |
| Suhuai | 21 | 18 | 3 | | | 0 | | | | 0.9286 | | | | | 0.0714 |
| Erhualian | 21 | 0 | 21 | | | 0 | | | | 0.5 | | | | | 0.5 |
| Mi | 20 | 0 | 20 | | | 0 | | | | 0.5 | | | | | 0.5 |
| Meishan | 19 | 0 | 19 | | | 0 | | | | 0.5 | | | | | 0.5 |
| PDLY | 50 | 37 | 13 | | | 0 | | | | 0.87 | | | | | 0.13 |
| Variant ID :SNV00010  g.-924 T/C | | Genotype | | | | | | | | Allele frequency | | | | | |
| Breed | Number | T/T | T/C | | | C/C | | | | T | | | | | C |
| Landrace | 22 | 8 | 13 | | | 1 | | | | 0.6591 | | | | | 0.3409 |
| Yorkshire | 19 | 17 | 2 | | | 0 | | | | 0.9474 | | | | | 0.0526 |
| Duroc | 7 | 7 | 0 | | | 0 | | | | 1 | | | | | 0 |
| Suhuai | 21 | 14 | 6 | | | 1 | | | | 0.8095 | | | | | 0.1905 |
| Erhualian | 21 | 0 | 0 | | | 21 | | | | 0 | | | | | 1 |
| Mi | 20 | 0 | 0 | | | 20 | | | | 0 | | | | | 1 |
| Meishan | 19 | 0 | 2 | | | 17 | | | | 0.0526 | | | | | 0.9474 |
| PDLY | 62 | 44 | 17 | | | 1 | | | | 0.8468 | | | | | 0.1532 |
| Variant ID :SNV00011  g.-930 C/A | | Genotype | | | | | | | | Allele frequency | | | | | |
| Breed | Number | C/C | C/A | | | A/A | | | | C | | | | | A |
| Landrace | 22 | 8 | 13 | | | 1 | | | | 0.6591 | | | | | 0.3409 |
| Yorkshire | 19 | 17 | 2 | | | 0 | | | | 0.9474 | | | | | 0.0526 |
| Duroc | 7 | 7 | 0 | | | 0 | | | | 1 | | | | | 0 |
| Suhuai | 21 | 14 | 6 | | | 1 | | | | 0.8095 | | | | | 0.1905 |
| Erhualian | 21 | 0 | 0 | | | 21 | | | | 0 | | | | | 1 |
| Mi | 20 | 0 | 0 | | | 20 | | | | 0 | | | | | 1 |
| Meishan | 19 | 0 | 2 | | | 17 | | | | 0.0526 | | | | | 0.9474 |
| PDLY | 62 | 44 | 17 | | | 1 | | | | 0.8468 | | | | | 0.1532 |
| Variant ID :SNV00012  g.-1143G/T | | Genotype | | | | | | | Allele frequency | | | | | | |
| Breed | Number | G/G | G/T | | | T/T | | | G | | | | | T | |
| Landrace | 22 | 18 | 4 | | | 0 | | | 0.9091 | | | | | 0.0909 | |
| Yorkshire | 19 | 18 | 0 | | | 1 | | | 0.9474 | | | | | 0.0526 | |
| Duroc | 7 | 7 | 0 | | | 0 | | | 1 | | | | | 0 | |
| Suhuai | 21 | 20 | 1 | | | 0 | | | 0.9762 | | | | | 0.0238 | |
| Erhualian | 21 | 21 | 0 | | | 0 | | | 1 | | | | | 0 | |
| Mi | 20 | 20 | 0 | | | 0 | | | 1 | | | | | 0 | |
| Meishan | 18 | 18 | 0 | | | 0 | | | 1 | | | | | 0 | |
| PDLY | 62 | 61 | 1 | | | 0 | | | 0.9919 | | | | | 0.0081 | |
| Variant ID :SNV00013  g.-1179C/G | | Genotype | | | | | | | Allele frequency | | | | | | |
| Breed | Number | C/C | C/G | | | G/G | | | C | | | | | G | |
| Landrace | 22 | 18 | 4 | | | 0 | | | 0.9091 | | | | | 0.0909 | |
| Yorkshire | 19 | 18 | 0 | | | 1 | | | 0.9474 | | | | | 0.0526 | |
| Duroc | 7 | 7 | 0 | | | 0 | | | 1 | | | | | 0 | |
| Suhuai | 20 | 19 | 1 | | | 0 | | | 0.975 | | | | | 0.025 | |
| Erhualian | 21 | 21 | 0 | | | 0 | | | 1 | | | | | 0 | |
| Mi | 20 | 20 | 0 | | | 0 | | | 1 | | | | | 0 | |
| Meishan | 15 | 15 | 0 | | | 0 | | | 1 | | | | | 0 | |
| PDLY | 52 | 52 | 0 | | | 0 | | | 1 | | | | | 0 | |
| Variant ID :SNV00014  g.-1421 A/G | | Genotype | | | | | | | Allele frequency | | | | | | |
| Breed | Number | A/A | A/G | | | G/G | | | A | | | | G | | |
| Landrace | 22 | 8 | 13 | | | 1 | | | 0.6591 | | | | 0.3409 | | |
| Yorkshire | 18 | 16 | 2 | | | 0 | | | 0.9444 | | | | 0.0556 | | |
| Duroc | 7 | 7 | 0 | | | 0 | | | 1 | | | | 0 | | |
| Suhuai | 21 | 14 | 6 | | | 1 | | | 0.8095 | | | | 0.1905 | | |
| Erhualian | 21 | 0 | 0 | | | 21 | | | 0 | | | | 1 | | |
| Mi | 20 | 0 | 0 | | | 20 | | | 0 | | | | 1 | | |
| Meishan | 19 | 0 | 2 | | | 17 | | | 0.0526 | | | | 0.9474 | | |
| PDLY | 50 | 36 | 13 | | | 1 | | | 0.85 | | | | 0.15 | | |
| Variant ID :SNV00015  g.-1448 AC/- | | Genotype | | | | | | | Allele frequency | | | | | | |
| Breed | Number | AC/AC | AC/- | | | -/- | | | AC | | | | - | | |
| Landrace | 22 | 7 | 15 | | | 0 | | | 0.6591 | | | | 0.3409 | | |
| Yorkshire | 19 | 1 | 18 | | | 0 | | | 0.5263 | | | | 0.4737 | | |
| Duroc | 7 | 0 | 7 | | | 0 | | | 0.5 | | | | 0.5 | | |
| Suhuai | 21 | 1 | 20 | | | 0 | | | 0.5238 | | | | 0.4762 | | |
| Erhualian | 21 | 21 | 0 | | | 0 | | | 1 | | | | 0 | | |
| Mi | 20 | 20 | 0 | | | 0 | | | 1 | | | | 0 | | |
| Meishan | 20 | 19 | 1 | | | 0 | | | 0.975 | | | | 0.025 | | |
| PDLY | 62 | 5 | 57 | | | 0 | | | 0.5403 | | | | 0.4597 | | |
| Variant ID :SNV00016  g.-1452 ACACAC/- | | Genotype | | | | | | | | | | Allele frequency | | | |
| Breed | Number | ACACAC/ACACAC | | | ACACAC/- | | | -/- | | | | ACACAC | | | - |
| Landrace | 22 | 16 | | | 6 | | | 0 | | | | 0.8636 | | | 0.1364 |
| Yorkshire | 19 | 19 | | | 0 | | | 0 | | | | 1 | | | 0 |
| Duroc | 7 | 7 | | | 0 | | | 0 | | | | 1 | | | 0 |
| Suhuai | 21 | 21 | | | 0 | | | 0 | | | | 1 | | | 0 |
| Erhualian | 21 | 21 | | | 0 | | | 0 | | | | 1 | | | 0 |
| Mi | 20 | 20 | | | 0 | | | 0 | | | | 1 | | | 0 |
| Meishan | 20 | 20 | | | 0 | | | 0 | | | | 1 | | | 0 |
| PDLY | 62 | 58 | | | 3 | | | 1 | | | | 0.9597 | | | 0.0403 |
| Variant ID :SNV00017  g.-1454 ACACACAC/- | | Genotype | | | | | | | | | Allele frequency | | | | |
| Breed | Number | ACACACAC/ACACAC | | ACACACAC/- | | | -/- | | | | ACACACAC | | | | - |
| Landrace | 22 | 22 | | 0 | | | 0 | | | | 1 | | | | 0 |
| Yorkshire | 19 | 19 | | 0 | | | 0 | | | | 1 | | | | 0 |
| Duroc | 7 | 7 | | 0 | | | 0 | | | | 1 | | | | 0 |
| Suhuai | 21 | 21 | | 0 | | | 0 | | | | 1 | | | | 0 |
| Erhualian | 21 | 21 | | 0 | | | 0 | | | | 1 | | | | 0 |
| Mi | 20 | 20 | | 0 | | | 0 | | | | 1 | | | | 0 |
| Meishan | 20 | 20 | | 0 | | | 0 | | | | 1 | | | | 0 |
| PDLY | 62 | 61 | | 0 | | | 1 | | | | 0.9839 | | | | 0.0161 |
| Variant ID :SNV00018  g.-1573 C/G | | Genotype | | | | | | | Allele frequency | | | | | | |
| Breed | Number | C/C | C/G | | | G/G | | | C | | | | G | | |
| Landrace | 22 | 9 | 12 | | | 1 | | | 0.6818 | | | | 0.3182 | | |
| Yorkshire | 19 | 17 | 2 | | | 0 | | | 0.9474 | | | | 0.0526 | | |
| Duroc | 7 | 7 | 0 | | | 0 | | | 1 | | | | 0 | | |
| Suhuai | 21 | 19 | 2 | | | 0 | | | 0.9524 | | | | 0.0476 | | |
| Erhualian | 21 | 10 | 11 | | | 0 | | | 0.7381 | | | | 0.2619 | | |
| Mi | 20 | 12 | 8 | | | 0 | | | 0.8 | | | | 0.2 | | |
| Meishan | 19 | 0 | 2 | | | 17 | | | 0.0526 | | | | 0.9474 | | |
| PDLY | 62 | 46 | 15 | | | 1 | | | 0.8629 | | | | 0.1371 | | |
