# Supplementary figures and images for: Comprehensive Analysis of Porcine Prox1 Gene and Its Relationship with Meat Quality Traits
Source: Animals (Basel). 2019 Sep 29;9(10):744. doi: 10.3390/ani9100744 (PMC6826434; doi:10.3390/ani9100744)

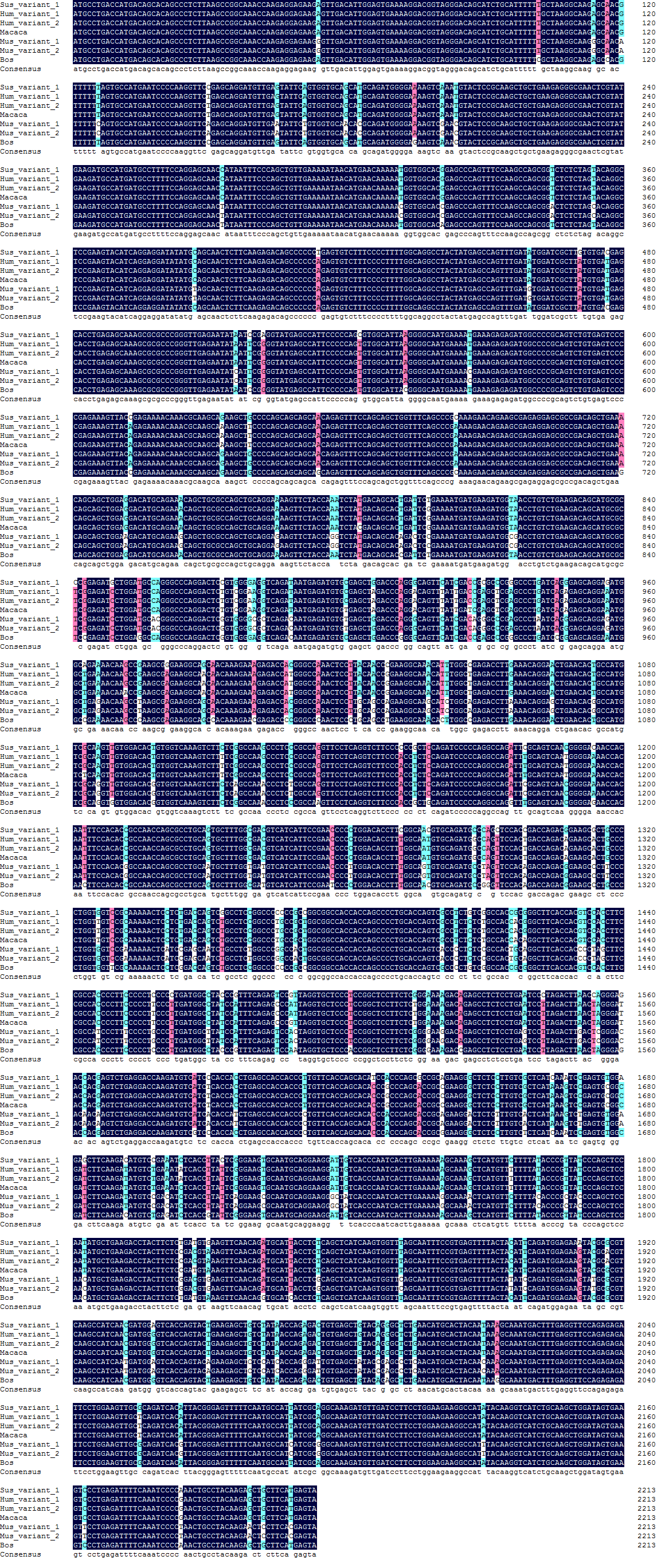

Supplement: Supplementary file 1 [file animals-09-00744-s001.zip › Supplementary Files/Figure S1.tif]

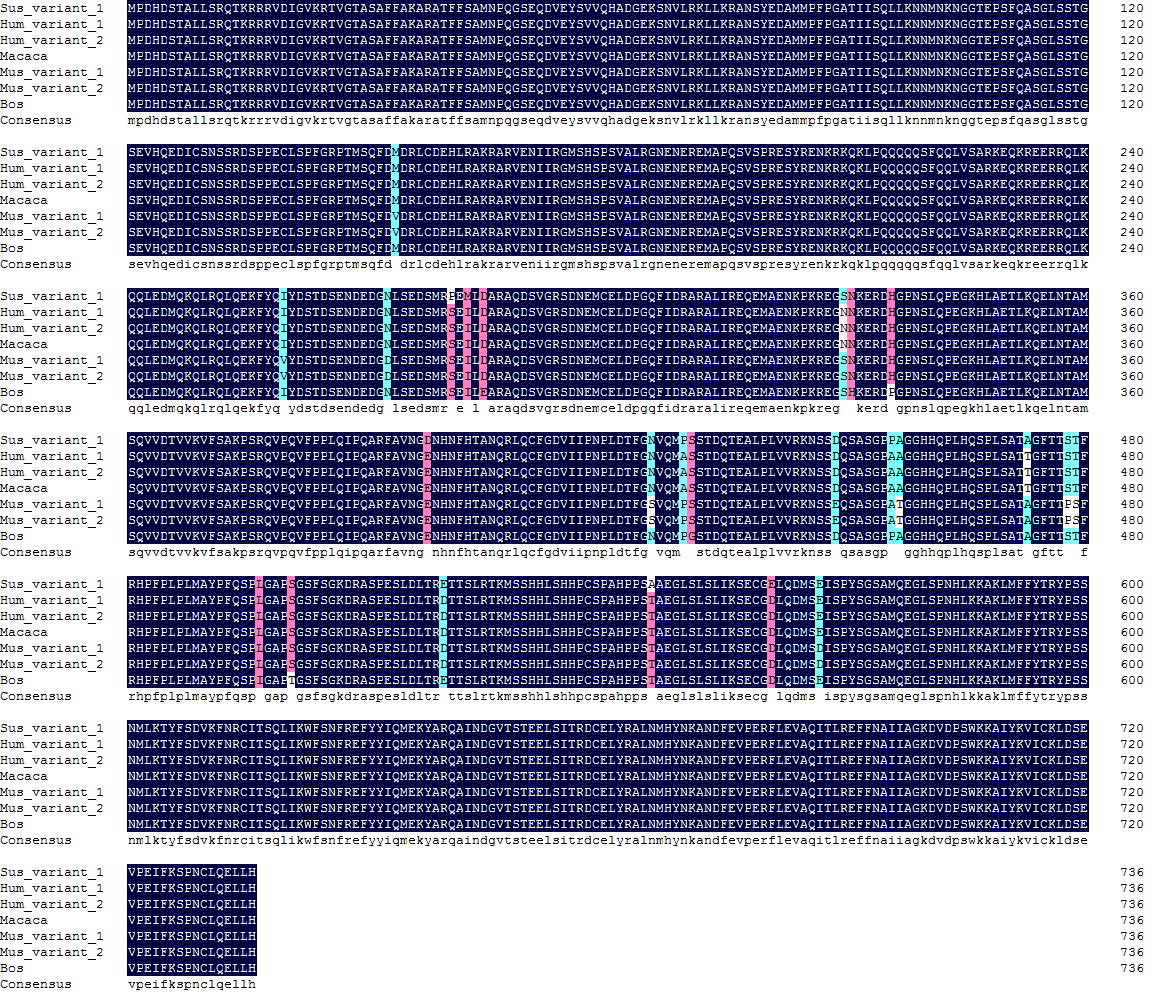

Supplement: Supplementary file 1 [file animals-09-00744-s001.zip › Supplementary Files/Figure S2.tif]

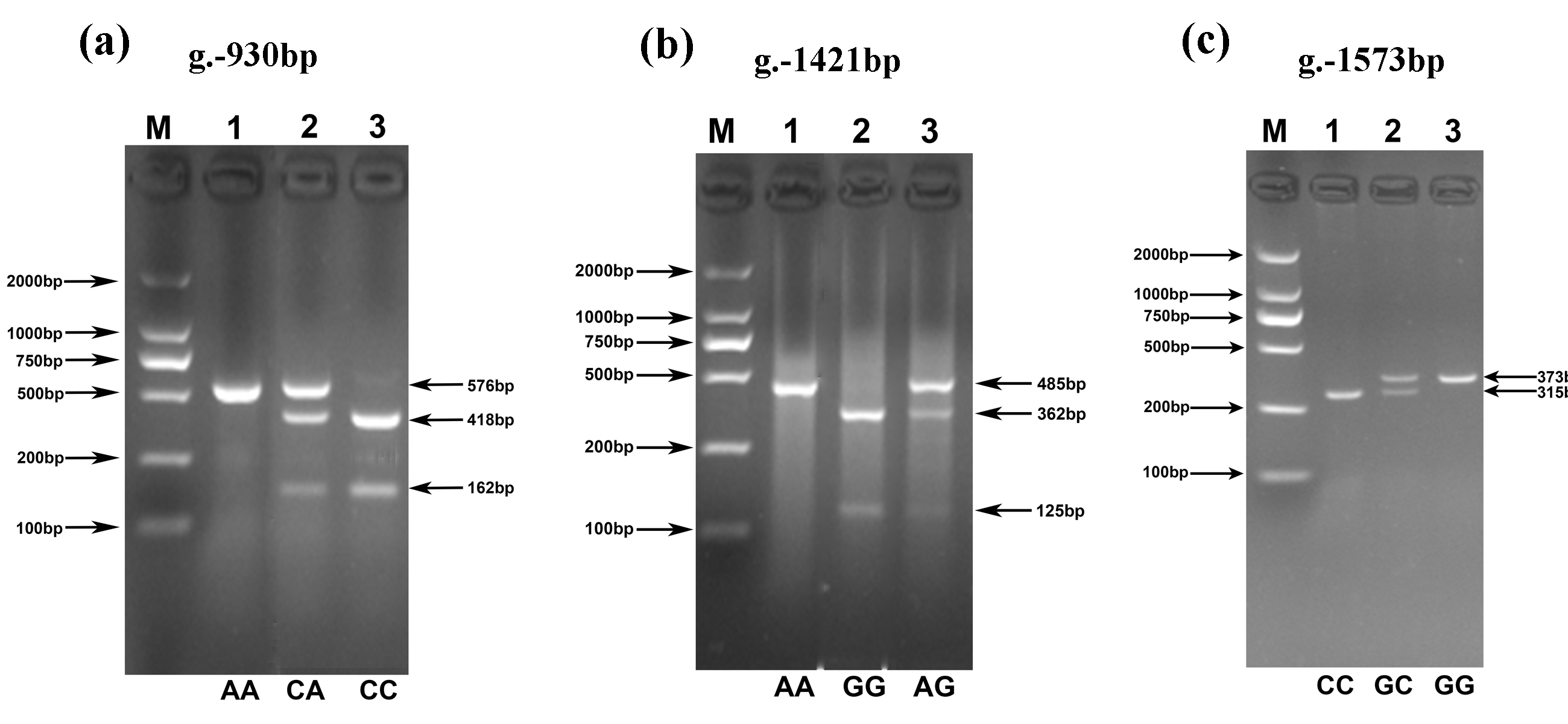

Supplement: Supplementary file 1 [file animals-09-00744-s001.zip › Supplementary Files/Figure S3.tif]
