# Supplementary material for: Comprehensive Analysis of Porcine Prox1 Gene and Its Relationship with Meat Quality Traits
Source: Animals (Basel). 2019 Sep 29;9(10):744. doi: 10.3390/ani9100744 (PMC6826434; doi:10.3390/ani9100744)
Supplement: Supplementary file 1 [file animals-09-00744-s001.zip › Supplementary Files/Table S1.doc]

Characteristics of Porcine *Prox1* Gene and Its Relationship with Production Traits.

**Chao Dong ^1,^** ^a^**, Yingxi Zhang ^1,^** ^a^**, Kaiqing Liu ^1^, Bojiang Li ^1, 2^, Zhe Chao ^3^, Aiwen Jiang ^1^, Rongyang Li ^1^, Zengkai Zhang ^1^, Pinghua Li ^1^, Honglin Liu ^1^ and Wangjun Wu ^1 +^**

**Supplementary Table S1 Primers used in this study.**

| **Primers used for amplification of porcine *Prox1* full-length cDNA** | | | | | | | | | | | | | |
| --- | --- | --- | --- | --- | --- | --- | --- | --- | --- | --- | --- | --- | --- |
| **Primer name^1^** | **Primer sequence（5’→3’）** | | | | | **Product size (bp)** | | | **Annealing**  **temperature (℃)** | | | | Application |
| GSP1 | gattacgccaagcttCCTGCCAAAAGGGGAAAGACACTCA | | | | | _ | | | _ | | | | 5’RACE first round PCR |
| NGSP1 | gattacgccaagcttCTGGGCTCCGTGCCACCATTTTTGT | | | | | _ | | | _ | | | | 5’RACE nested PCR |
| GSP2 | gattacgccaagcttATCATTCCGAACCCCCTGGACACCT | | | | | _ | | | _ | | | | 3’RACE first round PCR |
| NGSP2 | gattacgccaagcttGTCAAGCCATCAACGATGGAGTCAC | | | | | _ | | | _ | | | | 3’RACE nested PCR |
| C1F | AAGGCTCGGTCCCACTGCTCTCT | | | | | 1073 | | | 62 | | | | CDS |
| C1R | TCGGCTTGTTTTCTGCCATCTCC | | | | |  |  |  |  |  |  |  |  |
| C2F | GGGGCAATGAAAATGAAAGAGAG | | | | | 1076 | | | 60 | | | | CDS |
| C2R | TGGATGTGCTGGTGAACAAGGGT | | | | |  |  |  |  |  |  |  |  |
| C3F | CACGAGTCTGAGGACCAAGATGT | | | | | 822 | | | 56 | | | | CDS |
| C3R | AAACGGAAGAAAGTGAGAGGAAG | | | | |  |  |  |  |  |  |  |  |
| **Primers used for amplification of porcine *Prox1* promoter 1 region** | | | | | | | | | | | | | |
| Primer name | | | | | Primer sequence（5’→3’）^2^ | | | | | | | | Length (bp) |
| pGL3-1957/+318 | | | | | F：tctctatcgataggtacc**GAGCTC**TGATACATTTCGCAGAACC | | | | | | | | 2275 |
| pGL3-1182/+318 | | | | | F：tctctatcgataggtacc**GAGCTC**CCCGGGGAAAGAGACATTCT | | | | | | | | 1500 |
| pGL3-682/+318 | | | | | F：tctctatcgataggtacc**GAGCTC**ATTGTTTTCATCCCAAACCTACC | | | | | | | | 1000 |
| pGL3-192/+318 | | | | | F：tctctatcgataggtacc**GAGCTC**TCCTCTCCTCCCAGCCCC | | | | | | | | 510 |
| pGL3+122/+318 | | | | | F：tctctatcgataggtacc**GAGCTC**CTCTCCGCTTCGCTCCTCT | | | | | | | | 215 |
| pGL3+318 | | | | | R：ccaacagtaccggaatgcc**AAGCTT**ATAATTGGACCAGGGACGGTG | | | | | | | |  |
| **Primers used for amplification of porcine *Prox1* promoter 2 region** | | | | | | | | | | | | | |
| Primer name | | | | | Primer sequence（5’→3’）^3^ | | | | | | | | Length(bp) |
| pGL3-2178/+117 | | | | | F：tctctatcgataggtacc**GGTACC**GAATAATGGTCGTTGAGAGATG | | | | | | | | 2295 |
| pGL3-1584/+117 | | | | | F：tctctatcgataggtacc**GGTACC**GTTTCCTGTGGTGAGTCTGA | | | | | | | | 1701 |
| pGL3-1080/+117 | | | | | F：tctctatcgataggtacc**GGTACC**TTTTGGAAAGCTGATAGACAC | | | | | | | | 1197 |
| pGL3-458/+117 | | | | | F：tctctatcgataggtacc**GGTACC**TTACAGGTGAACCGAAAGAGA | | | | | | | | 575 |
| pGL3-146/+117 | | | | | F：tctctatcgataggtacc**GGTACC**GGAAGTTCATAGGCAGAGA | | | | | | | | 263 |
| pGL3+117 | | | | | R：ccaacagtaccggaatgcc**AAGCTT**AAACAATAAATTGAATGAGGCA | | | | | | | |  |
| **Primers used for gene expression** | | | | | | | | | | | | | |
| Primer name | | | | Primer sequence  (5’→3’) | | | | Product size (bp) | | | Annealing  temperature (℃) | | |
| *HPRT-*-F1  *HPRT-*R1 | | | | GGTCAAGCAGCATAATCCAAAG  CAAGGGCATAGCCTACCACAA | | | | 251 | | | 60 | | |
| *Prox1-*F2  *Prox1-*R2 | | | | CCGTTTCAGAGTCCGTTAGGT  TGGTGGGATGACATCTTGGTC | | | | 122 | | | 60 | | |
| *MyHC Ⅰ-*F3  *MyHC Ⅰ-*R3 | | | | CGACACACCTGTTGAGAAG  AGATGCGGATGCCCTCCA | | | | 233 | | | 60 | | |
| *MyHC ⅡB-*F4  *MyHC ⅡB-*R4 | | | | GTTCTGAAGAGGGTGGTAC  AGATGCGGATGCCCTCCA | | | | 234 | | | 60 | | |
| *MyHC ⅡX-*F5  *MyHC ⅡX-*R5 | | | | CTTCACTGGCGCAGCAGGT  AGATGCGGATGCCCTCCA | | | | 257 | | | 60 | | |
| *Myoglobin-*F6  *Myoglobin-*R6 | | | | GGATGAGATGAAGGCCTCTG  CCTCTCATGAACAAAGCAAC | | | | 437 | | | 60 | | |
|  | | | **Primers used for detection of variations in porcine *Prox1* promoter 1** | | | | | | | | |  | |
| Primer name | | | Primer sequence（5’→3’） | | | | | | | | | Product size | |
| *Prox1*_1-F  *Prox1*_1-R | | | TGCTGGGGCCGGGTACGTCAG  GAGAGGAGGAGAGGGAGGAGGAGGAGA | | | | | | | | | 234 bp | |
| *Prox1*_2-F  *Prox1*_2-R | | | GCGGCTGGGCGAGGGTGAG  GGCTCCGCTCCACAACAAGATTTACTTTAAGAC | | | | | | | | | 275 bp | |
| *Prox1*_3-F  *Prox1*_3-R | | | GCTCGGCCAACTTGATCAGCGGTATCTG  GGGCTGGGAGGAGAGGAGGGGAGAG | | | | | | | | | 273 bp | |
| *Prox1*_4-F  *Prox1*_4-R | | | TTCAAAGGAAGGGAGGATGACACGTATTCTTGA  CGATCGCTCTTCCAGGACGCAGGT | | | | | | | | | 273 bp | |
| *Prox1*_5-F  *Prox1*_5-R | | | CCAAACAACGCTGTCAGAGGTAA  TRGGGATGGCATCAGAGAAG | | | | | | | | | 292 bp | |
| *Prox1*_6-F  *Prox1*_6-R | | | GGCGTCAAAACTGTGTAAATCCT  GCCGGCTTTATTGCATCTGATATT | | | | | | | | | 254 bp | |
| *Prox1*_7-F  *Prox1*_7-R | | | TCCCSGGGAAAGAGACATTC  ACTCACCCCAGAATGATCAAAGAG | | | | | | | | | 289 bp | |
| *Prox1*_8-F  *Prox1*_8-R | | | GAGGGACACACGAAACACACTT  GCCCGCACGATTTCTTCAG | | | | | | | | | 272 bp | |
| *Prox1*_9-F  *Prox1*_9-R | | | TTTGRGACTGAAATTGTAAATGTCCT  GCGTTTATGACCTAAAGATCCTATAAAAGCTA | | | | | | | | | 288 bp | |
| *Prox1*_10-F  *Prox1*_10-R | | | AAACGAAGGCTGGGGGTGT  GRCCGACCACGACCACCAT | | | | | | | | | 275 bp | |
| *Prox1*_11-F  *Prox1*_11-R | | | CGAAAAGGACCCAACCAATAGAG  GGTTAGGTGTTGCGGATAAGAAAA | | | | | | | | | 269 bp | |
| *Prox1*_12-F  *Prox1*_12-R | | | CCCTGCCCTTCTCCCTTTT  AGAAATAATCTAGCAACGTGCCCATTAG | | | | | | | | | 286 bp | |
| **Primers used for PCR-RFLP genotyping** | | | | | | | | | | | | | |
| Primer name | | Sequences (5’→3’) | | | | | Annealing temperature | | | Product size | | | Restriction enzymes |
| SNV11-F  SNV11-R | | ATCTGAAGAAATCGTGCGGGC  AAGTGGGGGTTTGAGGGGTGG | | | | | 60℃ | | | 576 | | | *Taq*I |
| SNV14-F  SNV14-R | | TATTGGAAGTATGGGTGGTGT  CTTTTTTTGGTAGGATGGAGA | | | | | 60℃ | | | 485 | | | *Bse*NI |
| SNV18-F  SNV18-R | | GCCTGACTTTGTTTTTTCTTT  GCGAAGCAGAAACATTCATAC | | | | | 58℃ | | | 373 | | | *Hpa*II |

*Note*.^1^ GSP: Gene-Specific Primers; NGSP: Nested Gene-Specific Primers; Lowercase letters represent the additional sequence. ^2^ Protective bases are indicated by lowercase, and *Sac*I and *Hin*dIII restriction sites are marked by underline and bold type. ^3^ Protective bases are indicated by lowercase, and *Kpn*I and *Hin*dIII restriction sites are marked by underline and bold type.
